# Innovative Short Process of Preparation and Nitriding of Porous 316L Stainless Steel

**DOI:** 10.3390/ma18071564

**Published:** 2025-03-30

**Authors:** Chunheng Liu, Fang Zhang, Lei Zhang, Jun Peng, Hongtao Chang, Yongbin Wang

**Affiliations:** 1 School of Metallurgical Future Technology, Inner Mongolia University of Science and Technology, Baotou 014010, China; chunheng058@163.com (C.L.); pengjun@imust.edu.cn (J.P.); cht158@163.com (H.C.); wyb0313032@imust.edu.cn (Y.W.); 2 School of Rare Earth Industry, Inner Mongolia University of Science and Technology, Baotou 014010, China; 3 Key Laboratory of Green Extraction & Efficient Utilization of Light Rare-Earth Resources, Inner Mongolia University of Science and Technology, Ministry of Education, Baotou 014010, China; 4 Tenth Company of Inner Mongolia First Machinery Group Co., Ltd., Baotou 014030, China; 18604724273@163.com

**Keywords:** porous 316L stainless steel, vacuum sintering, gas nitriding, short process, mechanical properties

## Abstract

Porous 316L stainless steel has a low density and high specific surface area, and is easy to process due to the large number of pores within it, making it ideal for applications such as piping in the chemical and food industries, as a medical tool, or as a fuel cell pole plate material. Nitriding treatment can further improve the hardness and strength of porous stainless steel. In this paper, a method combining vacuum sintering and nitriding treatment was proposed, i.e., 316L stainless steel powder was used as the raw material, and porous 316L was sintered in a vacuum tube furnace, in which the porous stainless steel was nitrided with nitrogen gas during the cooling process. In the research process, thermodynamic calculation and differential thermal analysis were used to determine the optimum nitriding temperature range of 700 °C~850 °C and nitriding pressure of 0.4 MPa~0.8 MPa. With the increase in nitriding temperature and pressure, the nitrogen content in the sample increased, and the nitrogen content of porous 316L stainless steel after nitriding was 0.03%~0.86%. The results show that nitrogen exists exclusively in solid solution at nitriding temperatures of 700 °C and 750 °C. At nitriding temperatures of 800 °C and 850 °C, the nitrogen existed in both solid solution and chromium nitride (CrN), and the Vickers hardness at 0.08 MPa and 850 °C was 135 HV, which was 2.82 times higher than that before nitriding. The compressive strength of the specimens was maximum at a nitriding pressure of 0.04 MPa and 850 °C. The corrosion resistance of the specimens is optimized when the nitriding pressure is 0.04 MPa and the temperature is 800 °C.

## 1. Introduction

Porous metal materials exhibit unique properties distinct from those of dense materials, owing to the presence of pores. These characteristics include low density, high specific surface area, excellent energy absorption, low thermal conductivity, effective sound absorption, high permeability, electromagnetic wave absorption, thermal shock resistance, gas sensitivity, and recyclability [1,2,3,4,5,6,7]. Austenitic stainless steel (ASS) is utilized across a wide range of fields for its exceptional corrosion resistance, including pipelines in the chemical and food industries [8,9,10], tools and implants in medical technology [11], and metal bipolar plates in fuel cells [12]. Notably, porous 316 stainless steel exhibits high-temperature resistance, corrosion and oxidation resistance, superior mechanical properties, excellent biocompatibility, and ease of processing. As a result, it is extensively employed as both a structural and functional material in applications such as drug delivery implants, smart stainless steel materials, and fuel cells [13,14,15,16].

Nitrogen is an effective element for enhancing the strength, wear resistance, and corrosion resistance of materials. Furthermore, it serves as a robust stabilizer of the austenite phase in stainless steel. By incorporating nitrogen into austenitic stainless steel, the crystal structure of the steel is maintained, while its properties are significantly enhanced [17,18,19,20,21]. Nitriding methods typically include gas nitriding, liquid nitriding, ion nitriding, and so on [22,23]. Lakshmi Deepak Tadepalli et al. [24] found that nitriding treatment improved the wear resistance, hardness, and toughness of AISI 409 ferritic stainless steel, thereby broadening its range of applications. Ozgur Celik [25] demonstrated that low-temperature nitriding can increase the fatigue limit of titanium-stabilized AISI 321 austenitic stainless steel by about 15%, without affecting the ductility. Mustafa Yazici et al. [26] carried out plasma nitriding of various samples of AISI 316L stainless steel for 4 h at 400 °C. The conventional method of gas nitriding involves placing the workpiece in a sealed vessel and passing a nitrogenous gas (e.g., ammonia or nitrogen) at a certain pressure and for a specified time. Compared to gas nitriding, ion nitriding usually requires a longer processing time, higher investment in equipment, etc., which leads to higher production costs [27,28]. Although this method is relatively straightforward and can be easily adapted to various sample shapes, it necessitates a high level of seal integrity to prevent defects such as blackening or oxidation on the surface [29,30]. Implementing gas nitriding under vacuum conditions, where the nitrogen potential is elevated compared to conventional gas nitriding, enhances nitrogen diffusion, accelerates the nitriding rate, and reduces the nitriding cycle time, ultimately resulting in a cleaner surface. P. Hussin [31] reported that using a 1:1 ratio of ammonia to nitrogen for the nitriding of AISI 316L stainless steel led to an almost 50% increase in hardness without altering the grain structure. The nitrided samples exhibited superior surface hardness, residual stress, wear resistance, and resistance to magnetization compared to the unnitrided samples. However, research on nitriding methods for porous stainless steel remains limited [32,33,34]. Elevated nitriding temperatures enhance the nitrogen diffusion coefficient, significantly accelerating the nitrogen transport kinetics. When nitriding exceeds 700 °C, a pronounced increase in surface nitrogen concentration gradient drives rapid nitrogen migration toward the substrate, thereby enabling substantial reduction in nitriding duration to achieve target case depth [35,36]. This temperature-driven acceleration mechanism proved particularly advantageous for porous materials: their interconnected networks formed by pores facilitated the absorption of thermal stress inside the workpiece, thereby reducing the deformation of the workpiece and effectively mitigating structural deformation risks associated with conventional dense materials during high-temperature nitriding. Applying pressure enhanced the flowability and penetration effect of nitrogen in complex structures such as slits and deep holes. By increasing the gas pressure gradient, nitrogen more uniformly diffused into the interior of porous materials or the closed pores of the irregularly shaped workpiece, thus mitigating the issue of uneven nitriding layer thickness [37,38].

Due to the high porosity, large specific surface area, and favorable interface reaction conditions of porous stainless steel, gas nitriding presents a distinct advantage over the treatment of dense metal devices. In this study, 316L stainless steel powder was initially subjected to vacuum sintering in a tube furnace. Then high-purity nitrogen was introduced into the furnace when the tube furnace was cooled down to nitriding temperature. The objective of this method is to enhance the mechanical properties and corrosion resistance of porous 316L stainless steel, thereby expanding the potential applications of this material. It is anticipated that the process for porous stainless steel will enable the integration of vacuum sintering and gas nitriding, thus streamlining the process flow, saving production time, ensuring product quality, and reducing production costs. Moreover, nitriding under vacuum conditions precludes the destruction of the nitriding environment by oxygen in the air, thereby ensuring the purity of the material. Additionally, nitrogen can more effectively penetrate into the porous material, thus guaranteeing the uniform thickness and chemical composition of the percolated layer. 

## 2. Materials and Methods

### 2.1. Experimental Materials

**Figure 1:**
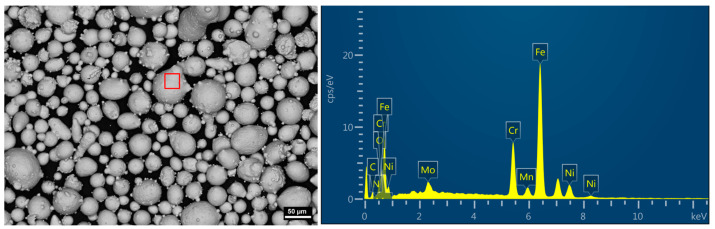
SEM-EDS analysis results of 316L stainless steel powder.

The primary material used in this study is 316L stainless steel powder, with its chemical composition detailed in Table 1. As illustrated in Figure 1, the results of the SEM-EDS analysis of 316L stainless steel powder are presented.The powder particles have a size of less than 150 mesh. For the nitriding process, high-purity nitrogen gas (purity ≥ 99.99%) is utilized as the nitrogen source.

### 2.2. Research Methods

Firstly, the occurrence forms of nitrogen in 316L stainless steel under varying temperatures and pressures were conducted using FactSage 8.1 thermodynamic software. The equilibrium module was employed, utilizing the FactPS, FSstel, and FToxid databases. The calculations were performed on a 100 g sample based on the chemical compositions listed in Table 1. Additionally, the states of nitrogen presence in 316L stainless steel under different temperatures and nitrogen pressures were investigated within a closed system.

**Figure 2:**
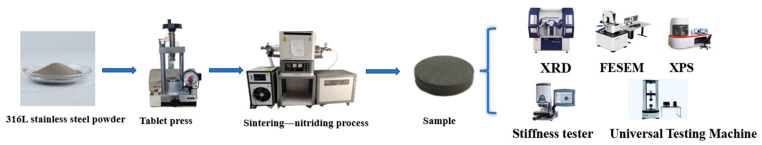
The research technical route of preparation and nitriding of porous 316L stainless steel.

Figure 2 depicts the process flow diagram of the experiment. Subsequently, the samples of porous 316 stainless steel were prepared by compression. A tablet press was employed to compress 12 g of 316L stainless steel powder at a constant pressure of 4 MPa for 2 min, creating flat moldings samples. Prior to the pressing of the specimen, less than 1 g of binder (NH_4_HCO_3_ Gel) is employed on the powder to be compressed, thereby facilitating its adhesion.

The flat moldings samples were placed in a controllable atmosphere vacuum tube furnace (SK-G08163, Tianjin, China) for sintering and nitriding. Sintering was conducted at 1200 °C for 90 min under a vacuum of 10–3 Pa. Following this, the samples were cooled to the nitriding temperature, at which point high-purity nitrogen gas was introduced at a specified pressure and maintained for 30 min. The samples were then cooled to room temperature before removal.

The nitrogen content of the nitrided specimens was quantified using an ONH-2000 inert gas fusion analyzer (Elementar, Germany), following ASTM E1019 standards. Prior to analysis, the specimens were crushed into irregular fragments (nominal dimensions < 10 mm) to ensure homogeneity. The nitrogen content was finally calculated. The temperature range for the formation of nitrides in 316L powder was determined using a high-temperature thermogravimetric analyzer (SETSYS Evolution, Setaram, Lyon, France). The phase composition of the nitrided samples was analyzed with an X-ray diffractometer (MiniFlex 600, Rigaku, Tokyo Metropolis, Japan). The microstructure and local composition of the nitrided samples were examined using a scanning electron microscope (SEM, SUPRA 55 FESEM, Carl Zeiss, Jena, Germany) and an energy-dispersive X-ray spectroscopy (EDS) system (X-MAX 20, Oxford, Oxford, UK). X-ray photoelectron spectroscopy (XPS, Escalab 250Xi, East Grinstead, UK) was employed to investigate the nitrogen states under various processing conditions. The surface microhardness of the nitrided samples was determined following the procedures outlined in GB/T 4340.1-2009 Vickers hardness test for metallic materials Part 1: Test methods. To test the hardness properties of nitrided porous stainless steel, the Vickers hardness of the specimen surface was measured using a Q10A+ fully automatic microhardness tester(Shanghai Hengyi Precision Instrument Co., Ltd, Shanghai, China) with a load of 9.8 N (1 kg) and a loading time of 15 s. Due to the unevenness of the porous stainless steel surface, the indentation tests were repeated 30 times under the same conditions to ensure that the data were reasonable. The maximum and minimum values were then excluded from the remaining data and all approximate averages were used. The porosity of the specimen was determined using the immersion medium method. The porosity of porous stainless steel can be tested by referring to “Permeable Sintered Metallic Materials—Determination of Open Porosity”. The basic principle is the Archimedes drainage method, which selects anhydrous ethanol as the immersion medium and determines the porosity of the specimen at room temperature.

**Figure 3:**
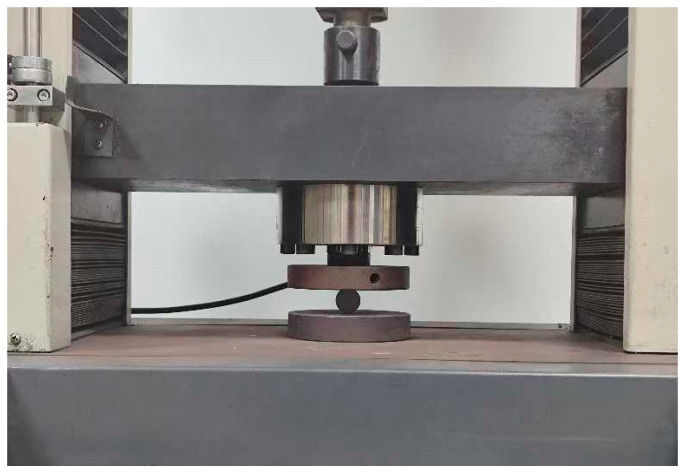
Compressive testing equipment for samples.

The compressive strength was evaluated using a universal testing machine (CMT4105, SANSYS, Shenzhen, China). Due to the flat moldings of the samples, they were initially positioned flat on the testing table, as illustrated in Figure 3. A constant force of 3 KN was applied downward to the nitrided samples, and the radial compressive deformation of the samples was recorded to assess their compressive strength. In the absence of cracking, a smaller deformation indicated a higher compressive strength.

The experiments were conducted using a conventional three-electrode system with the experimental specimen as the working electrode, a platinum sheet as the counter electrode, and a saturated calomel electrode (SCE) as the reference electrode, in 3.5 wt.% NaCl solution compliant with ASTM G64-86 (2018). The specimens were machined to 10 mm × 10 mm working surfaces, epoxy-encapsulated on non-working areas, and polished after curing to ensure full electrolyte contact. Polarization curves were acquired via linear sweep voltammetry at 0.1667 mV/s, while electrochemical impedance spectroscopy (EIS) measurements covered a frequency range of 104 to 5 × 10^−2^ Hz. All data were analyzed using Zview 2.7 software.

## 3. Determination of Nitriding Process

**Figure 4:**
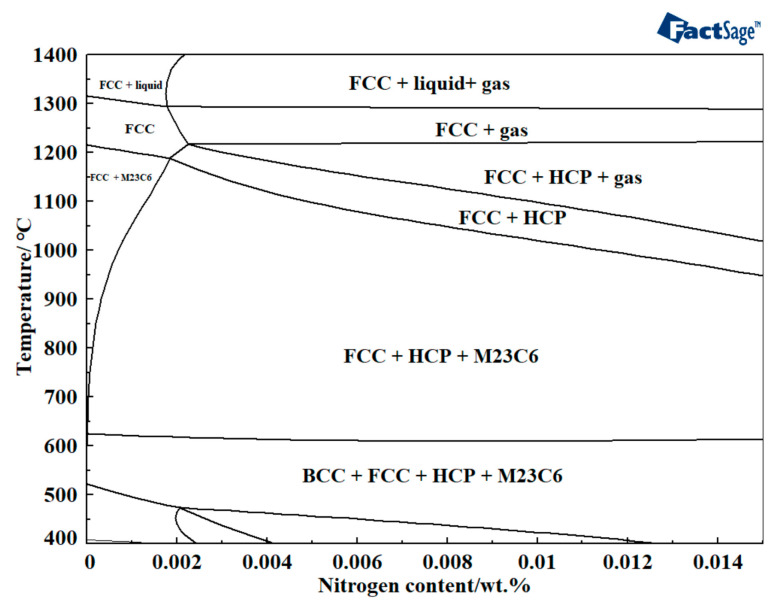
Pseudo-binary phase diagram phase diagram of Fe-N (17% Cr-12% Ni-2.5% Mo-0.03% C).

The FactSage 8.1 thermodynamic database equilibrium module was employed to calculate the nitriding temperature for porous stainless steel within a closed system. Figure 4 illustrates the Fe-N (17%Cr-12%Ni-2.5%Mo-0.03%C) binary phase diagram. The diagram illustrates that as the nitrogen content increases, M23C6 and HCP phases precipitate from FCC phase. FCC is a face-cubic structure formed when the sintering temperature is sufficiently high and the content of each element is above the critical level for stabilizing austenite. Given that HCP was a hexagonal carbon–nitride compound with a complex crystal structure and fewer slip systems, which created chromium (Cr) and molybdenum (Mo) depletion zones around it, this adversely affected the material’s overall mechanical properties and corrosion resistance [39,40,41]. At temperatures exceeding 625 °C, the presence of these three phases was evident. Conversely, the BCC phase also precipitated when the temperature was below 625 °C. BCC exhibits a body-centered cubic structure, resulting from low-temperature sintering at lower temperatures and inadequate elemental diffusion. Therefore, selecting the appropriate nitriding temperature was critical to the nitriding process.

**Figure 5:**
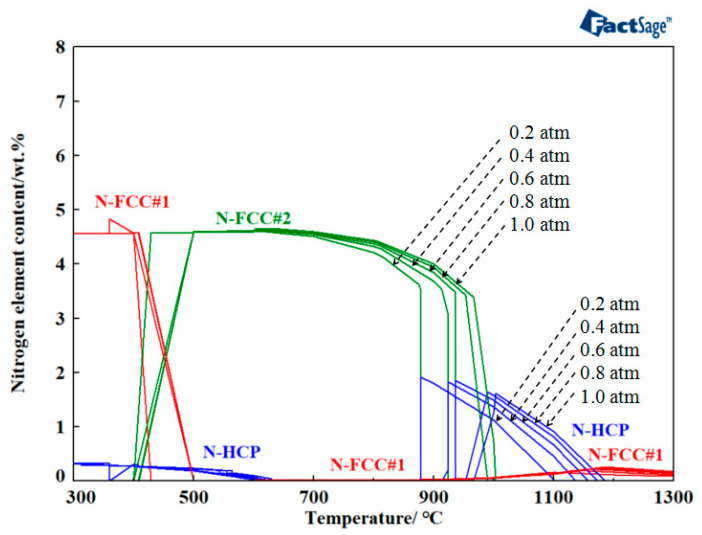
Distribution of nitrogen in different temperatures and pressure conditions.

The nitrogen contents in 316L stainless steel under varying system pressures and temperatures were calculated using FactSage 8.1, along with the chemical compositions outlined in Table 1, as illustrated in Figure 5. As shown in Figure 5, the emergence of both the FCC#1 and HCP phases in 316L stainless steel in nitrogen gas occurred at first. FCC#1 is raw austenite (γ-Fe) An austenitic matrix with no nitriding or low nitrogen content, stabilized in low-temperature-sintered 316L stainless steel. When nitriding is performed, nitrogen atoms are solidly dissolved in the austenite lattice interstices, forming FCC#2 (supersaturated solid solution). At 400 °C, the FCC#2 phase appeared and gradually reached a maximum of 4.65 wt.%. At 630 °C, the FCC#1 and HCP phases were completely eliminated, and by 879 °C, the FCC#2 phase were also eradicated, leading to the reappearance of the FCC#1 and HCP phases. Therefore, for system pressures ranging from 0.2 to 1.0 atmospheres, the optimal nitriding temperatures were decided between 700 and 850 °C.

**Figure 6:**
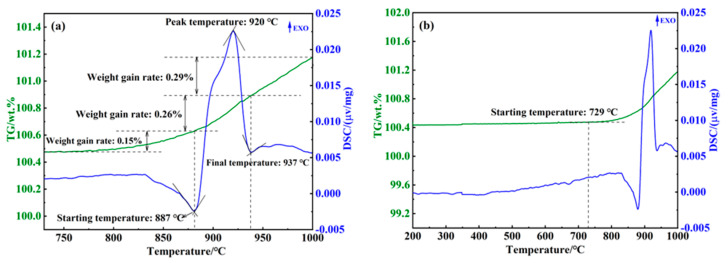
TG-DSC analysis of nitriding in porous 316L stainless steel. (**a**) 729 °C Post system weight gain trend (**b**) TG-DSC trends throughout the warming process.

To determine the generation temperature of the nitriding reaction, 316L stainless steel powder was analyzed using thermogravimetric differential scanning calorimetry (TG-DSC) with a differential thermal analyzer, as shown in Figure 6. Figure 6a indicates that weight gain on the thermogravimetric (TG) curve began at 729 °C, reaching a total of 0.70 wt.% by 1000 °C. Figure 6b illustrates that exothermic peaks on the differential scanning calorimetry (DSC) curve occurred between 887 °C and 937 °C, corresponding to a weight gain of 0.26 wt.%. A weight gain of 0.12% was observed in the temperature range of 729 °C to 887 °C, while 0.26 wt.% was gained between 887 °C and 937 °C, and 0.29 wt.% was gained from 937 °C to the end of the heating process. The weight gain rate observed in the interval between 729 °C and 887 °C was lower compared to the range above 887 °C. Therefore, it can be concluded that the nitridation of nitrogen with 316L stainless steel occurs between 887 °C and 937 °C, with nitrogen atoms effectively dissolving into the porous 316L matrix from 775 °C to 887 °C. Consequently, selecting a nitriding temperature below 850 °C, according Figure 5, was a reasonable approach.

**Figure 7:**
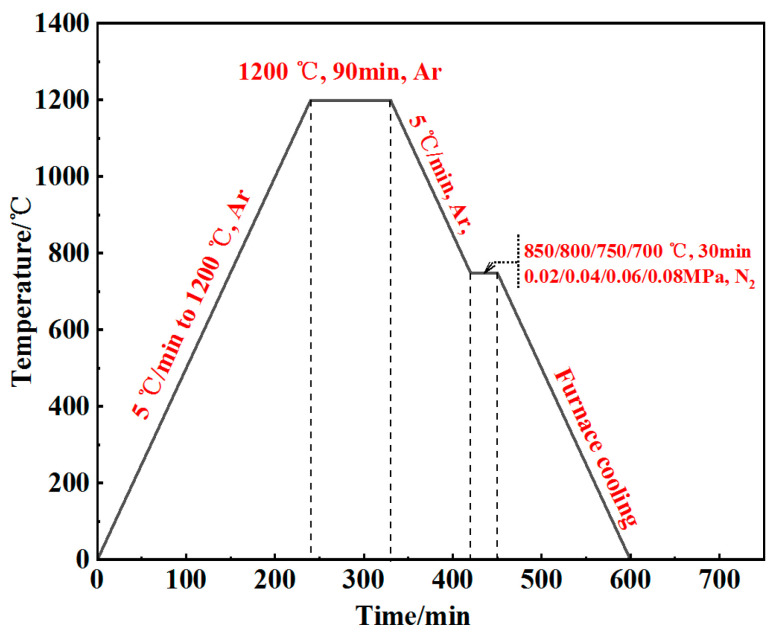
Sintering and nitriding process route for porous 316L stainless steel.

Figure 7 illustrates the sintering and nitriding procedures for porous 316L stainless steel. The sintering process was conducted at 1200 °C for 60 min in an argon atmosphere at a pressure of 0.1 atm. The nitriding temperatures were set at 700 °C, 750 °C, 800 °C, and 850 °C, each with a hold time of 30 min and high-purity nitrogen pressures of 0.2 atm, 0.4 atm, 0.6 atm, and 0.8 atm, respectively. The duration of the nitriding process significantly influenced the depth of the nitriding layer; prolonged nitriding times led to deeper layers. However, extended nitriding durations resulted in the formation of alloy nitrides, which diminished the corrosion resistance of the sample [42,43]. In the system of pure nitrogen, it is necessary to introduce a kinetic model of surface adsorption–dissociation.

$$K_{N}=\frac{P_{N_{2}}}{P_{0}}e^{\frac{-E_{a}}{k_{B}T}}$$

$P_{N_{2}}$: nitrogen partial pressure
$E_{a}$: nitrogen atom separation activation energy
k_B_: Boltzmann’s constant
T: absolute temperature (K)

**Figure 8:**
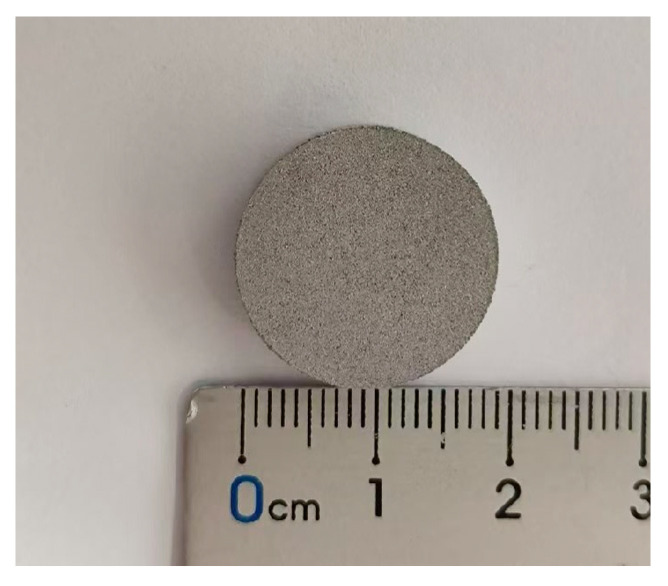
Macromorphology of porous 316L stainless steel flat molding after sintering–nitriding.

The dissociation of a nitrogen molecule (N_2_) into a reactive nitrogen atom (N*) requires the overcoming of activation energy. An increase in temperature results in a decrease in the absolute value of the exponent, thereby leading to a substantial increase in Kn. Furthermore, an increase in the partial pressure of nitrogen also results in an increase in the value of Kn. Figure 8 depicts the physical appearance of the samples after sintering and nitriding.

**Figure 9:**
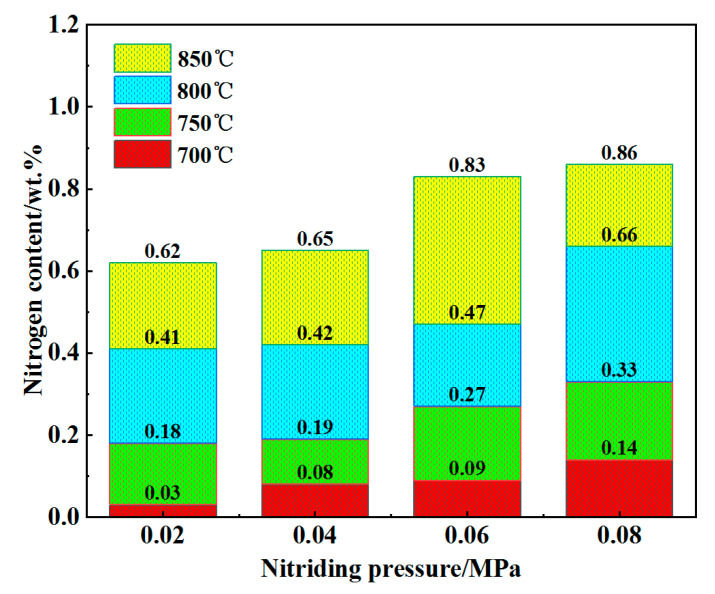
Nitrogen content in porous 316L stainless steel samples after nitriding.

Figure 9 illustrates the nitrogen content in porous 316L stainless steel under various nitriding conditions. As shown in the figure, the nitrogen content of the nitrided samples exhibited a significant increase, rising from 0.03 wt.% to 0.86 wt.%. The nitrogen content was observed to increase with rising nitriding pressure at a constant nitriding temperature. Similarly, at a constant nitriding pressure, the nitrogen content increased with higher nitriding temperatures. For nitriding pressures ranging from 0.02 MPa to 0.08 MPa and temperatures of 700 °C, 750 °C, 800 °C, and 850 °C, the nitrogen content increased by 0.11 wt.%, 0.57 wt.%, 0.74 wt.%, and 0.72 wt.%, respectively. Therefore, it can be concluded that at nitriding pressures above 0.06 MPa, the increase in nitrogen content was more pronounced at elevated temperatures. This phenomenon attributed to the nitriding process occurring within a sealed tube furnace. Higher system pressures facilitated the introduction of greater quantities of nitrogen gas, thereby increasing the concentration of nitrogen per unit volume. This resulted in a higher frequency of nitrogen atom collisions with the sample, enhancing the diffusion of nitrogen atoms into the material [44,45].

## 4. Effects of Nitriding on Properties of Porous 316L Stainless Steel

### 4.1. Phase Composition of Nitrided Samples

**Figure 10:**
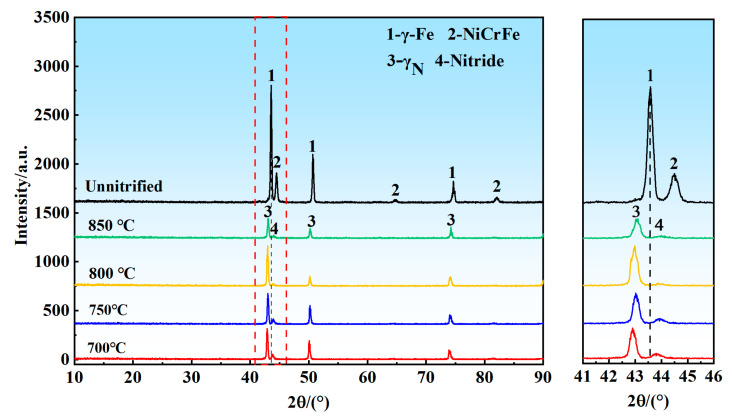
XRD patterns of nitrided porous 316L stainless steel samples at 0.02 MPa.

**Figure 11:**
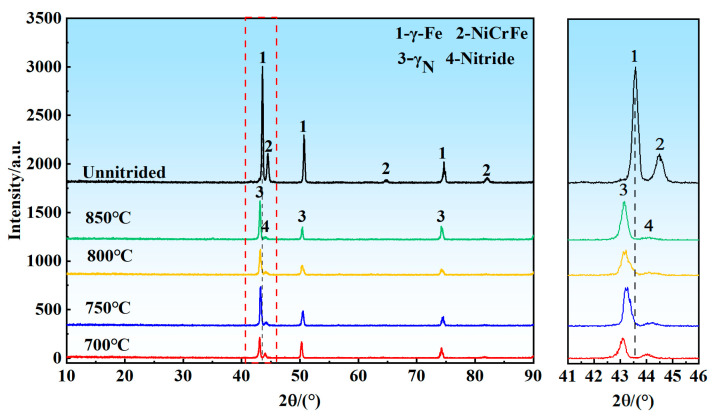
XRD patterns of nitrided porous 316L stainless steel samples at 0.08 MPa.

Figure 10 and Figure 11 illustrate the phase compositions of porous 316L stainless steel under pressures of 0.02 MPa and 0.08 MPa, respectively. The data presented in both figures indicate that the primary phases present in the porous 316L stainless steel were austenite (γ-Fe) and NiCrFe phases prior to nitriding. Following nitriding, the diffraction peaks of austenite exhibited a slight shift to the left, indicating the formation of nitrogen-containing austenite. This shift suggested that nitrogen dissolved into the austenite lattice, resulting in lattice distortion and expansion, which caused the increase in interplanar spacing [46,47,48]. Furthermore, as shown in the figures, the nitrides formed in 316L porous after nitriding. Additional analysis is necessary to determine the chemical composition and molecular structure of these nitrides, which can be accomplished through supplementary detection methods.

### 4.2. Forms of Nitrogen Presence

**Figure 12:**
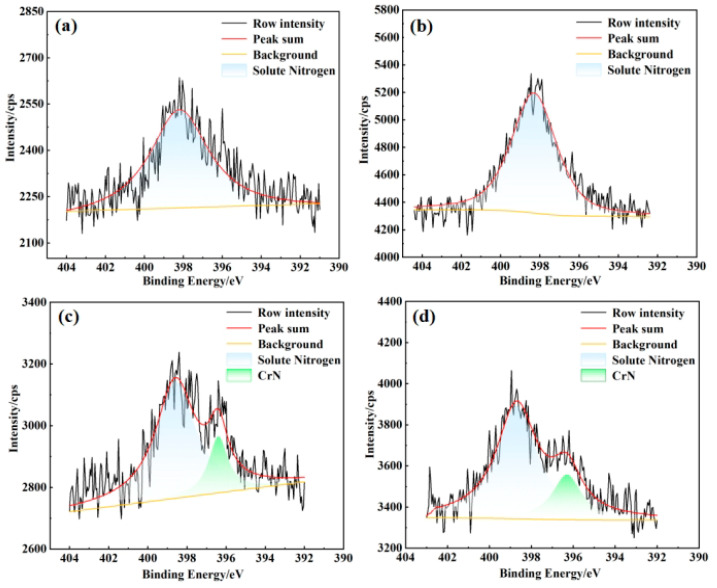
XPS analysis of nitrogen in nitrided porous 316L stainless steel at 0.02 MPa, (**a**) 700 °C; (**b**) 750 °C; (**c**) 800 °C; and (**d**) 850 °C.

**Figure 13:**
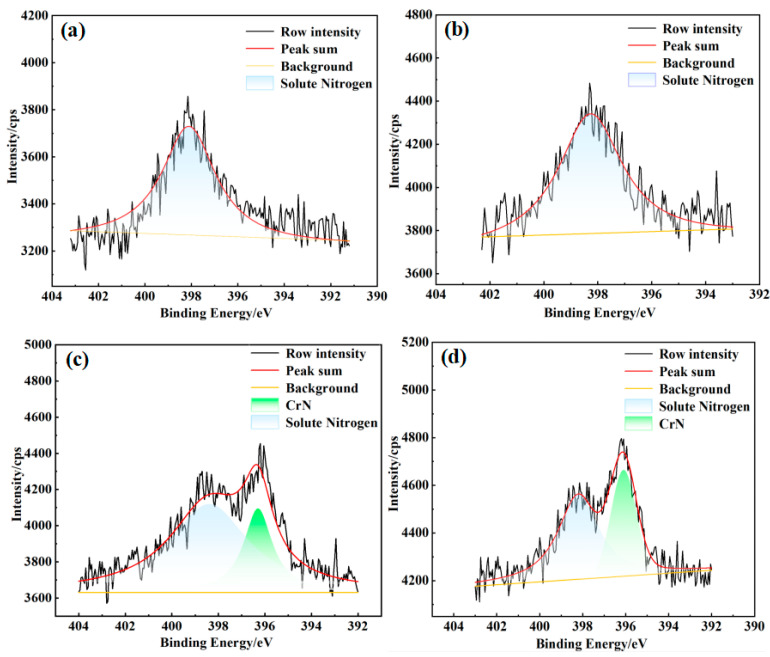
XPS analysis of nitrogen in nitrided porous 316L stainless steel at 0.08 MPa. (**a**) 700 °C; (**b**) 750 °C; (**c**) 800 °C; and (**d**) 850 °C.

To investigate the forms of nitrogen in nitrided samples under varying conditions, X-ray photoelectron spectroscopy (XPS) peak fitting and analysis were conducted. Figure 12 and Figure 13 present the XPS spectra for nitriding at temperatures of 700 °C and 750 °C under nitrogen pressures of 0.02 MPa and 0.08 MPa. The XPS peaks for the nitrogen-containing substance in porous 316L stainless steel fell within the binding energy range of 396~400 eV. Specifically, a binding energy of 396.6 ± 0.1 eV corresponded to chromium nitride (CrN), 397.2 ± 0.1 eV to chromium dinitride (Cr2N), and 398.1 ± 0.1 eV to solid solution nitrogen [49].

As illustrated in Figure 12 and Figure 13, at nitrogen pressures of 0.02 MPa and 0.08 MPa, and nitriding temperatures of 700 °C and 750 °C, nitrogen was predominantly present in a solid solution form [50]. At elevated temperatures of 800 °C and 850 °C, nitrogen existed in both solid solution form and as chromium nitrides. The X-ray diffraction (XRD) results presented in Figure 10 and Figure 11 suggested the probable occurrence of nitrides in the states of CrN or Cr_2_N. Thermodynamic calculations further indicated that at temperatures of 800 °C and 850 °C, the nitrides were primarily CrN, whereas at temperatures exceeding 879 °C, the nitrides in the precipitated hexagonal close-packed (HCP) phase were Cr_2_N. Therefore, it can be concluded that, at pressures of 0.04 MPa and 0.06 MPa, and temperatures of 700 °C and 750 °C, nitrogen was present as a solid solution. Additionally, at 800 °C and 850 °C, it existed as both solid solution nitrogen and chromium nitrides.

Furthermore, in the nitriding process of porous 316L stainless steel, the nitriding temperature had a direct impact on the chemical state of nitrogen. In contrast, variations in nitriding pressure had a minimal effect on the distribution of nitrogen within the samples. This is because the nitriding temperature significantly influences the nitrogen content. When the nitrogen content exceeded the solubility limit for the austenite phase of 316L stainless steel, it was precipitated in the form of nitrides. As illustrated in Figure 9, the formation of chromium nitride (CrN) occurred when the nitrogen content in porous 316L stainless steel exceeded 0.40 wt.%.

### 4.3. Microscopic Morphology of Nitrided Samples

**Figure 14:**
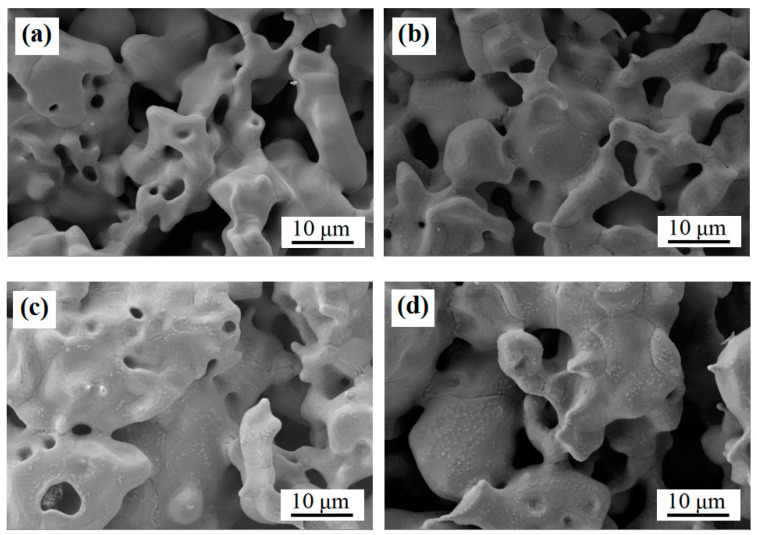
Microscopic morphology of nitrided sample surfaces at 0.02 MPa and various temperatures. (**a**) 700 °C; (**b**) 750 °C; (**c**) 800 °C; and (**d**) 850 °C.

Figure 14 presents the surface microtopography of nitrided specimens under 0.02 MPa at varying temperatures. The specimens exhibit irregularly shaped pore structures characterized by smooth peripheral edges and distinct sintering necks. The porosity of the specimens was measured to be within the range of 36% to 38% by means of Archimedes’ method in all the processes. The nitriding process exerted minimal influence on the porosity of the specimens. At elevated temperatures of 800 °C and 850 °C, particulate precipitates are observed on the substrate surface. These precipitates display heterogeneous size distributions yet maintain well-defined interfaces with the substrate. Notably, the absence of a discernible diffusion layer suggests strong interfacial bonding. Localized regions reveal fine precipitates (likely CrN or Fe3N in their initial nucleation stages), while grain boundary-aligned precipitates confirm preferential nitride nucleation at these sites. In contrast, specimens processed at 700 °C and 750 °C exhibit smoother substrate surfaces. The temperature-dependent emergence of granular precipitates correlates with nitrogen supersaturation levels, strongly implying that such features originate from nitride formation when nitrogen concentrations exceed solid solution solubility limits.

**Figure 15:**
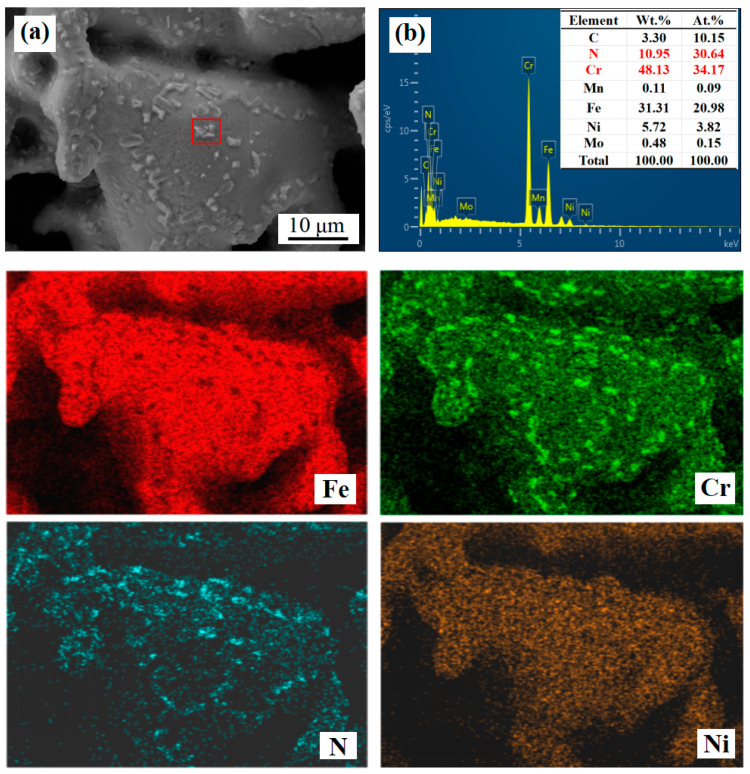
SEM-EDS and elemental distribution of surface precipitates in nitrided samples at 0.02 MPa and 850 °C. (**a**) Map of particles precipitated on the surface of the porous layer (**b**) SEM-EDS analysis of particles in red box.

A scanning electron microscope equipped with energy-dispersive spectroscopy (SEM-EDS) was employed to analyze the surface precipitates of nitrided samples at a pressure of 0.02 MPa and a temperature of 800 °C, as shown in Figure 15. The analysis reveals that the precipitates contain elevated levels of nitrogen (10.95 wt.%) and chromium (48.13 wt.%). The spatial distribution maps for nitrogen and chromium demonstrated a significant degree of overlap, confirming that the surface precipitates were nitrides. In conjunction with the results from X-ray photoelectron spectroscopy (XPS), it can be concluded that the precipitated nitrides were chromium nitride (CrN).

**Figure 16:**
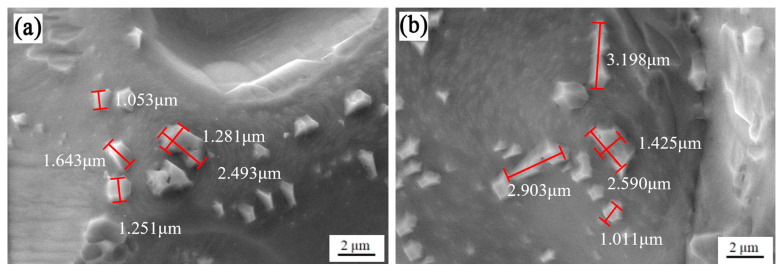
Microscopic morphology of nitrides in samples nitrided at 0.02 MPa; (**a**) 800 °C; and (**b**) 850 °C.

As illustrated in Figure 16, microstructural and morphological analyses were conducted on chromium nitride (CrN) precipitates formed at the surface of a specimen nitrided under 0.02 MPa. The figure demonstrates that equiaxed crystalline CrN particles are embedded within the matrix surface, with labeled dimensions indicating their size distribution. The largest CrN particle measures 3.12 μm, while smaller particles are observed at 1.05 μm, yielding an average particle size of approximately 2.01 μm. These finer precipitates are progressively nucleating and growing through dynamic precipitation processes. Elevated nitriding temperatures provide essential activation energy for CrN formation and growth, effectively overcoming interfacial free energy barriers to initiate nucleation while simultaneously promoting precipitate segregation and coarsening [51,52,53,54,55].

## 5. Mechanical Properties of Porous 316L Stainless Steel After Nitriding

### 5.1. Vickers Hardness

**Figure 17:**
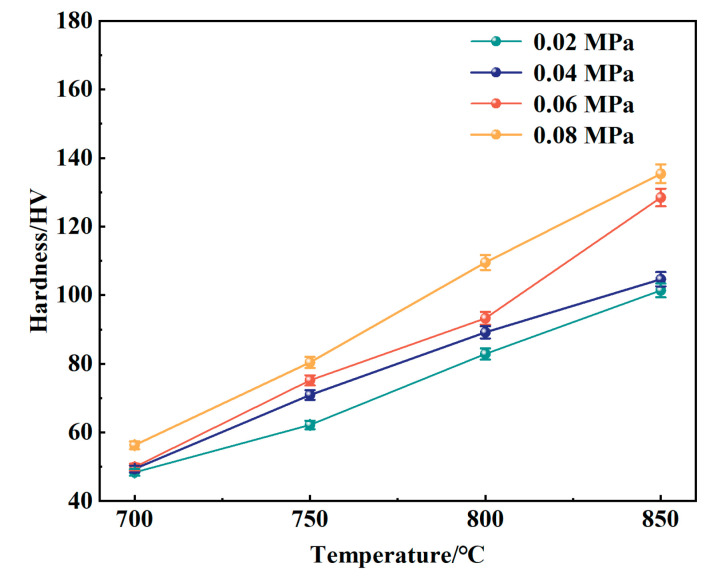
Microscopic hardness of nitrided samples.

Figure 17 illustrates the Vickers hardness of the samples before and after nitriding. The Vickers hardness of the 316L porous stainless steel prior to nitriding was measured at 47.88 HV. Following nitriding, an increase in hardness was typically observed with rising nitriding temperatures. At a pressure of 0.02 MPa, the hardness at 700 °C was 48.13 HV, indicating only a slight increase compared to the unnitrided sample. However, at 850 °C, the hardness increases to 101.37 HV, which was 2.12 times greater than that of the unnitrided sample. This increase can be attributed to the nitrogen dissolved in the austenitic structure, which causes lattice expansion and distortion, thereby elevating dislocation energy and enhancing surface hardness [56]. At a nitriding pressure of 0.08 MPa, the hardness of the specimen at 850 °C reached 135 HV, which was 2.82 times that of the specimen prior to nitriding. This increase in hardness was due to the presence of solid-state nitrogen within the matrix, as well as excess nitrogen precipitated at the grain boundaries and within the crystal lattice. The precipitation of chromium and nitrogen elements resulted in elevated dislocation energy when the samples were subjected to external forces, significantly enhancing the hardness [57].

### 5.2. Compressive Strength

**Figure 18:**
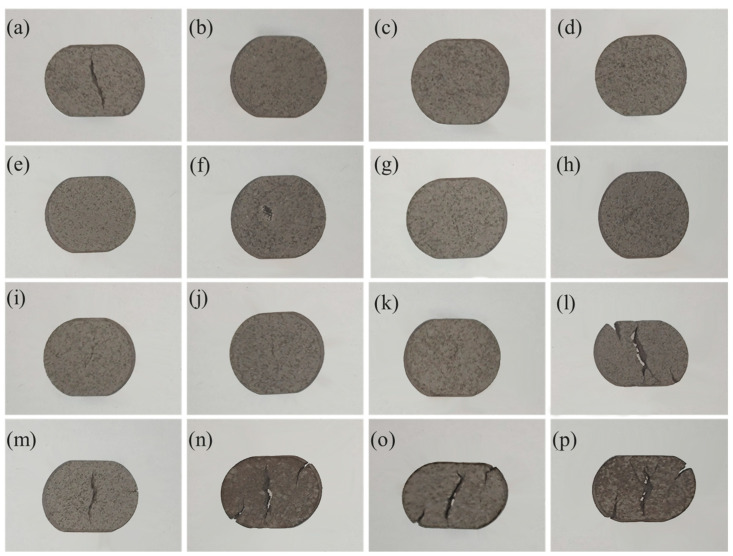
Samples compressed under identical pressure conditions. (**a**–**d**) 0.02 MPa; (**a**) 700 °C; (**b**) 750 °Ca; (**c**) 800 °C; (**d**) 850 °C; (**e**–**h**) 0.04 MPa; (**e**) 700 °C; (**f**) 750 °C; (**g**) 800 °C; (**h**) 850 °C; (**i**–**l**) 0.06 MPa; (**i**) 700 °C; (**j**) 750 °C; (**k**) 800 °C; (**l**) 850 °C; (**m**–**p**) 0.08 MPa; (**m**) 700 °C; (**n**) 750 °Ca; (**o**) 800 °C; and (**p**) 850 °C.

Figure 18 presents a photographic representation of the nitrided samples subjected to a pressure of 3 kN at varying pressures and temperatures. As illustrated in the figure, at 0.02 MPa, the sample nitrided at 700 °C exhibited vertical cracking, while the samples nitrided at 750 °C, 800 °C, and 850 °C showed no cracks but experienced vertical shrinkage. The extent of shrinkage was observed to decrease with increasing temperature. At 0.04 MPa, no cracks were detected at any temperature; however, vertical shrinkage was evident, with the degree of shrinkage diminishing as the temperature rose. At 0.06 MPa, minor fissures appeared in the center of the nitrided samples at 700 °C, 750 °C, and 800 °C. These fissures became increasingly pronounced at higher temperatures, reaching a significant level at 850 °C. At 0.08 MPa, only the sample nitrided at 700 °C did not exhibit any cracks, while the other samples displayed noticeable cracks at both the edges and the center. As the temperature increased, the cracks became more pronounced.

**Figure 19:**
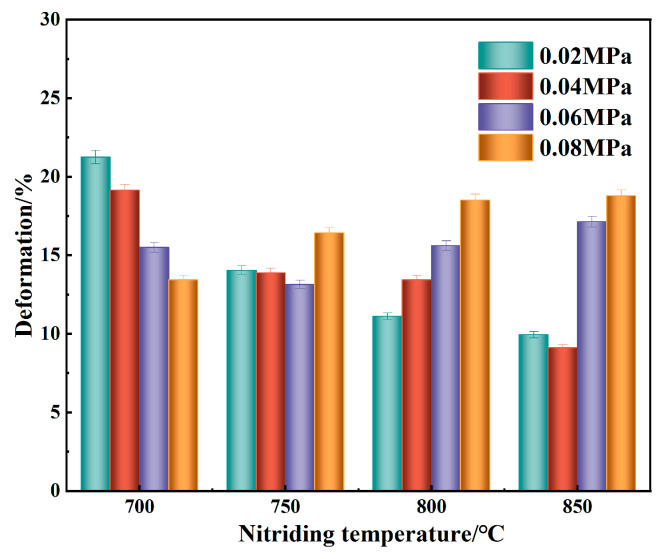
Deformation of porous 316L stainless steel nitriding samples at the same pressure.

Figure 19 illustrates the deformation of porous 316L stainless steel samples following nitriding. As shown, at nitriding pressures of 0.02 MPa and 0.04 MPa, the deformation of the nitrided samples decreased with increasing nitriding temperature. At 0.06 MPa, deformation initially decreases before increasing as the temperature rose. In contrast, at 0.08 MPa, deformation consistently increases with higher nitriding temperatures. At 0.02 MPa and 0.04 MPa, the increase in nitrogen content resulted in a higher concentration of solid solution nitrogen in the matrix, which enhances compressive strength through solid solution strengthening [58]. However, under high-temperature and high-pressure nitriding conditions, the formation of nitrides and changes in microstructure, such as the transformation of austenite into other phases, can negatively impact mechanical properties, leading to reduced compressive strength [59]. The lowest deformation and the highest compressive strength were observed at a nitriding pressure of 0.04 MPa and a temperature of 850 °C.

### 5.3. Electrochemical Analysis of Nitrided Specimens

**Figure 20:**
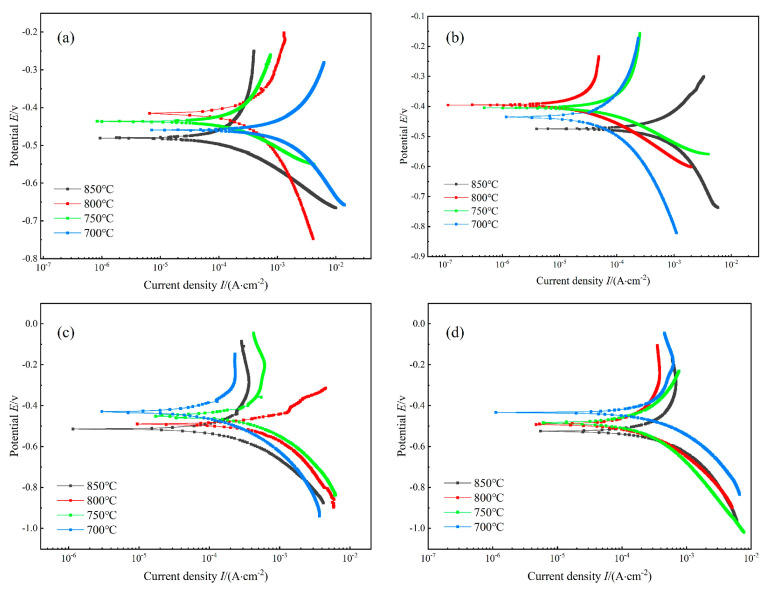
Polarization curves of nitriding specimens with different nitriding temperatures. (**a**) 0.02 MPa, (**b**) 0.04 MPa, (**c**) 0.06 MPa, and (**d**) 0.08 MPa.

As illustrated in Figure 20, the polarization curves of nitrided specimens in a 3.5% NaCl electrolyte are shown under various nitriding conditions. It is evident from Figure 20 that at nitriding pressures of 0.02 MPa and 0.04 MPa, the self-corrosion potential of the specimens exhibits an initial decline followed by an increase with the rise in nitriding temperature. Conversely, at nitriding pressures of 0.06 MPa and 0.08 MPa, the self-corrosion potential of the specimens demonstrates a rise with an increase in nitriding temperature. In different nitriding pressure conditions at 850 °C, the reason for the corrosion of specimens with the smallest self-corrosion potential is that nitrogen atoms diffuse into the matrix during the nitriding process at high temperatures. This diffusion occurs beyond the solid solubility of the matrix, forming nitride precipitation. This results in chromium depletion in the vicinity of the precipitates. The formation of corrosion micro-areas during the corrosion process also reduces the corrosion resistance of the specimen.

As shown in Table 2, the corrosion potential (Ecorr) initially increased but later decreased with rising nitriding pressure (0.02 MPa), while higher nitriding temperatures generally elevated Ecorr. The lowest Ecorr (−0.412 V) and corrosion current density (Icorr = 1.46 × 10^−4^ A/cm^2^) were observed at 800 °C under 0.02 MPa, indicating optimal corrosion resistance. However, at 800 °C and 0.04 MPa, Ecorr slightly improved to −0.396 V with a significantly lower Icorr (0.27 × 10^−4^ A/cm^2^), suggesting an enhanced corrosion resistance. For a nitriding pressure of 0.06 MPa, Ecorr increased steadily with temperature. At 700 °C, Ecorr reached its minimum (−0.430 V) with Icorr = 0.80 × 10^−4^ A/cm^2^, demonstrating minimal corrosion susceptibility. Conversely, at lower pressures (e.g., 0.08 MPa), Ecorr at 700 °C was −0.434 V (Icorr = 1.18 × 10^−4^ A/cm^2^), further confirming the improved resistance.

Overall, Ecorr varied moderately with nitriding temperature. The optimal corrosion resistance occurred at 0.04 MPa and 800 °C, exhibiting the highest Ecorr and lowest Icorr. A comparative ranking of corrosion resistance was established: 0.04 MPa/800 °C > 0.02 MPa/800 °C > 0.06 MPa/700 °C > 0.08 MPa/700 °C. Notably, the post-nitriding Ecorr values (−0.396 to −0.434 V) significantly surpassed the pre-nitriding baseline (−0.497 V) [60], confirming that appropriate nitriding parameters effectively enhance corrosion resistance.

**Figure 21:**
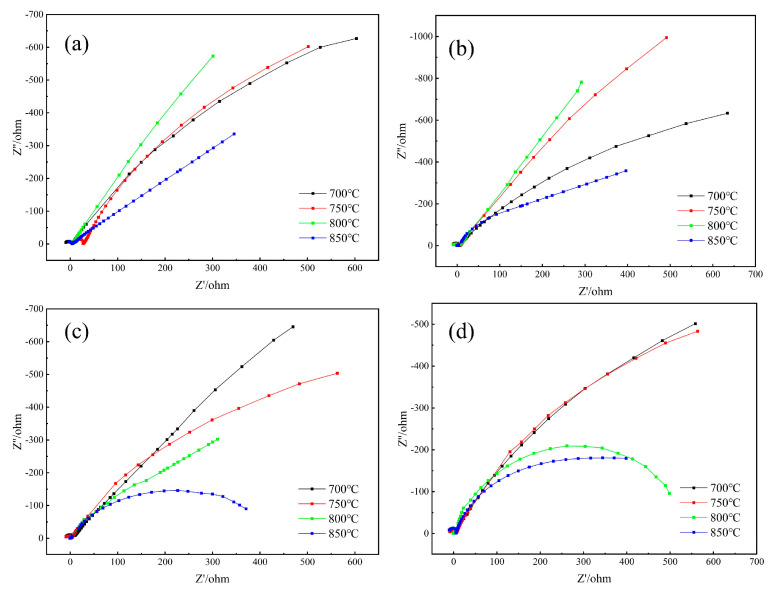
Nyquist plots of nitrided specimens at different nitriding temperatures. (**a**) 0.02 MPa, (**b**) 0.04 MPa, (**c**) 0.06 MPa, and (**d**) 0.08 MPa.

**Figure 22:**
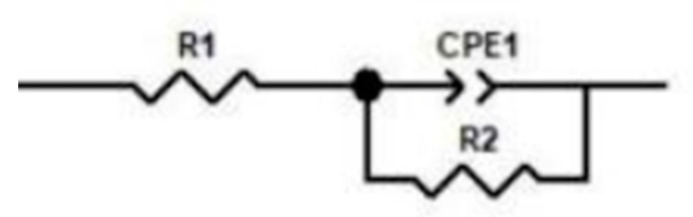
The equivalent circuit diagram of the impedance.

As shown in Figure 21, the impedance spectra of nitrided specimens under varying temperatures exhibit distinct profiles, consisting of high-frequency capacitive semicircles and low-frequency diffusion tails. These spectra were fitted using the equivalent circuit in Figure 22, where polarization impedance magnitude directly correlates with corrosion resistance. Specifically, higher charge transfer resistance R2 corresponds to superior corrosion inhibition [61], reflecting the protective efficacy of the nitrided layer. In 3.5 wt.% NaCl solution, solution resistance R1 remained stable across all conditions, while R2 values varied significantly. Specimens nitrided at 800 °C under 0.02–0.04 MPa exhibited the largest R2 radii, indicating optimal corrosion resistance. Conversely, at higher pressures (0.06–0.08 MPa), specimens nitrided at 700 °C showed enhanced R2 values (Figure 21). This behavior aligns with the nitride precipitation characteristics discussed. A continuous nitrided layer effectively blocks corrosive media infiltration, improving substrate protection. However, excessive nitride formation at elevated temperatures/pressures induces chromium depletion zones, accelerating localized corrosion and reducing overall resistance.

## 6. Conclusions

To suppress the formation of the hexagonal close-packed (HCP) phase and promote the development of single-phase austenite, the optimal nitriding temperature for porous 316L stainless steel was determined to be between 700 °C and 850 °C. The nitriding pressures varied from 0.02 MPa to 0.08 MPa. After a 30 min holding period at these temperatures, the lowest nitrogen content recorded was 0.03 wt.% at 700 °C and 0.02 MPa, while the highest content observed was 0.86 wt.% at 850 °C and 0.08 MPa. The nitrogen content was significantly influenced by the nitriding temperature.

The phase composition of the nitrided porous 316L stainless steel samples was predominantly γ_N_. At nitriding temperatures of 700 °C and 750 °C, nitrogen was primarily present as a solid solution. However, at nitriding temperatures of 800 °C and 850 °C, nitrogen existed in both a solid solution and the CrN phase. At these elevated temperatures, the nitrided samples also exhibited equiaxed CrN precipitates embedded within the matrix surface.

The Vickers hardness and compressive strength of the nitrided samples generally increased with rising nitriding temperatures. Under nitriding conditions of 0.08 MPa and 850 °C, the Vickers hardness reached 135 HV, which was 2.82 times higher than that of the samples prior to nitriding. The sample nitrided at 0.04 MPa and 850 °C exhibitd the lowest degree of deformation and the highest compressive strength.

Electrochemical testing in 3.5 wt.% NaCl solution revealed that specimens nitrided at 0.04 MPa and 800 °C exhibited superior corrosion resistance, with the highest corrosion potential (Ecorr = −0.396 V) and the lowest corrosion current density (Icorr = 0.27 × 10^−4^ A/cm^2^). These results confirm that harsher nitriding conditions enhance corrosion resistance by optimizing the protective nitrided layer, effectively delaying substrate degradation.

## Figures and Tables

**Table 1:** Chemical Composition of Porous 316L Stainless Steel, wt.%.

C	Cr	Ni	Mo	S	P	N	Fe
0.025	16.790	12.000	2.490	0.015	0.020	0.000	Bal.

**Table 2:** Parameters obtained from polarization curve fitting.

Nitriding Temperatures/°C	0.02 MPa		0.04 MPa		0.06 MPa		0.08 MPa	
	Ecorr/v	Icorr/(A/cm^2^)	Ecorr/v	Icorr/(A/cm^2^)	Ecorr/v	Icorr/(A/cm^2^)	Ecorr/v	Icorr/(A/cm^2^)
700	−0.460	0.001220	−0.435	0.000046	−0.430	0.000080	−0.434	0.000118
750	−0.436	0.000192	−0.405	0.000078	−0.452	0.000249	−0.486	0.000120
800	−0.412	0.000146	−0.396	0.000027	−0.490	0.000542	−0.492	0.000211
850	−0.481	0.000283	−0.475	0.000530	−0.515	0.000137	−0.525	0.000237

## Data Availability

The original contributions presented in this study are included in the article. Further inquiries can be directed to the corresponding author.
